# The Relationship between Calcaneal Spur Type and Plantar Fasciitis in Chinese Population

**DOI:** 10.1155/2020/5679629

**Published:** 2020-06-04

**Authors:** Lei Zhang, Han-wen Cheng, Lu-jing Xiong, Zhang-rong Xia, Meng-yao Zhang, Shi-jie Fu, Guo-you Wang

**Affiliations:** ^1^Department of Orthopedics, Affiliated Traditional Chinese Medicine Hospital of Southwest Medical University, Luzhou, China; ^2^Center for Orthopedic Diseases Research, Affiliated Traditional Chinese Medicine Hospital of Southwest Medical University, China; ^3^Clinical Base of Affiliated Traditional Chinese Medicine Hospital of Southwest Medical University, Guangdong Province Medical 3D Printing Application Transformation Engineering Technology Research Center, China; ^4^Academician Workstation Construction Project of Luzhou, Sichuan Province, China; ^5^School of Clinical Medicine, Southwest Medical University, Luzhou, China

## Abstract

Plantar heel pain is a common disease with a high incidence in different races. It significantly reduced the quality of life of patients. However, the cause of PHP is still controversial and there were varieties of physiological factors associated with PHP. The most common pathological factor in the population was plantar fasciitis. Some existing research studies had found a correlation between calcaneal spurs and plantar fasciitis, and this study had found the correlation in Chinese population. It is invaluable not only to understand the relationship between different types of plantar calcaneal spurs and plantar fasciitis but also to identify the most appropriate treatment strategies. A total of 71 patients with calcaneal spurs were chosen from the Affiliated Traditional Chinese Medicine Hospital of Southwest Medical University. All 71 patients had completed X-rays and MRI scans; then, surgeons had removed their plantar calcaneal spurs. After surgery, all patients were followed up for 12 months; their prognosis was tested by the VAS and AOFAS scores. Type II (29, 40.8%) had the highest incidence in Chinese population, followed with type I (24, 33.8%) and type III (18, 25.4%). Preoperative VAS scores showed that type II (7.72 ± 1.10) was significantly higher than the other two types (*P* < 0.001). Postoperative VAS scores of type II were higher than those of type I and type III (*P* < 0.001). Postoperative AOFAS scores of type II were the lowest (*P* < 0.001). Researchers had proved that type II was more likely to cause PF.

## 1. Introduction

Plantar heel pain (PHP) is a common disease with a high incidence in different races [1–3]. It was expected to cost more than 200 million of dollars in PHP treatment [4]. In addition, PHP had greatly affected the quality of life of patients as well as foot weight-bearing functions such as walking and standing [5, 6]. The cause of PHP is still controversial, and there were varieties of physiological factors associated with PHP [7–9]; the most common pathological factor in population was plantar fasciitis (PF) [10, 11]. However, whether the plantar calcaneal spur (PCS) had directly caused the PF remained unclear [12, 13]. There were a number of studies that suggested a link between PCS and PF [12, 14]. This study was focused on the relationship between several different types of PCS and PF.

Research studies on the PF and classification of PCS had a long tradition [2, 13, 15], but there were little about the relationship between PCS and PF in Chinese population. Some research studies suggested that PF should be a degenerative disease or fascia disease rather than an inflammatory disease [10]. Others research studies had shown that the old people, obese people, and arthritic people were more likely to develop PF [16–18]. Moreover, histological research studies had found that PF had a fascia fragmentation and degeneration [9], which may be caused by PCS at the plantar bottom [19, 20]. Therefore, the interests of researchers in the study of PCS and PF were aroused. Most PCS derived from the calcaneal tuberosity and formed on the posterior plantar surface of the calcaneus [21]. There were different forms of PCS, and they could be divided into two or three types now [13, 22]. Recently, Böddeker et al. had proved that PCS originated from endochondral and soft tissue ossification [23, 24]. Abreu et al. had confirmed plantar fascia thickening was related to the formation of PCS [25, 26], and Yi et al. had found that the thickening of plantar fascia was closely related to the occurrence of PF [27]. Furthermore, Li and Muehleman had directly pointed out that PF may be associated with PCS [28]. Zhou et al. had classified PCS into two types and found that the severity of PF caused by different types of PCS was significantly different [13]. Later, Ahmad and Karim had found that both the horizontal and hooked spurs showed the greatest improvement in function and pain after treatment [22]. Although surgery was thought to have a better therapeutic effect, the effect ultimately depended on the cause of PHP [29]. At present, there was no clear description of different types of PCS patient treatment strategies in Chinese population.

It was invaluable to understand the relationship between each type of PCS and PF in Chinese population. This study classified Chinese PCS into three types based on the angle between the calcaneal spur and the calcaneus. Researchers had also measured the morphological parameters of PCS which may affect the occurrence of PF. At the same time, researchers observed the severity of PF in each patient by magnetic resonance imaging (MRI) results, recording the visual analog scale (VAS) and the American Orthopedic Foot and Ankle Society (AOFAS) scores of each patient. Finally, researchers had proved the relationship between PCS type and PF in Chinese population, which could instruct surgeons to choose a more appropriate treatment.

## 2. Materials and Methods

### 2.1. Ethical Statement

Ethical approval was given by the medical ethics committee of Southwest Medical University with the following reference number KY2018012.

### 2.2. Materials

A total of 71 patients with calcaneal spurs were chosen from the Affiliated Traditional Chinese Medicine Hospital of Southwest Medical University. This study was conducted between January 2017 and January 2019. All patients were Chinese with varying degrees of plantar fascia degeneration and PHP for more than one year. Patients with congenital calcaneal deformities and calcaneal fractures were excluded. 71 patients contained 34 left calcaneus (17 males, 17 females) and 37 right calcaneus (18 males, 19 females). The average age of 34 patients was 42.5 (range 31 to 67), and the average age of 37 patients was 47.8 (range 35 to 65). All patients had completed the X-ray and MRI examinations; then, surgeons had removed their PCS.

### 2.3. Methods for Classification of PCS

**Figure 1:**
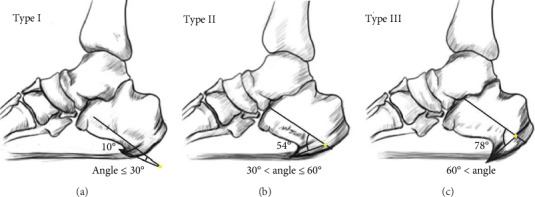
Hand-drawn diagram of classification of PCS. Type I, angle was less than 30° (a). Type II, angle was from 30° to 60° (b). Type III, angle was more than 60° (c).

**Figure 2:**
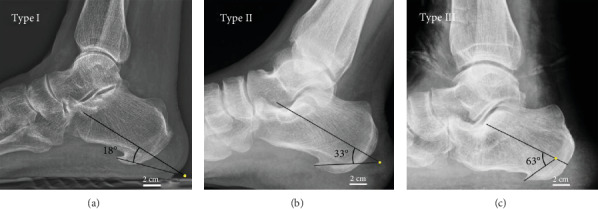
X-rays of classification of PCS. Type I, angle was less than 30° (a). Type II, angle was from 30° to 60° (b). Type III, angle was more than 60° (c).

X-ray was used to measure the angle between the PCS and the calcaneus. The angle between the PCS and the calcaneus was measured by a protractor (Model 6002, provided by Zhejiang Chute Co., Ltd.). A CT three-dimensional reconstruction scanner was used to reconstruct and capture the PCS' morphology. The classification was based on the angle between the PCS and the calcaneus. The direction of one line is parallel to the central axis of the calcaneus, and another one is parallel to the central axis of the PCS. According to the angle between the PCS and the calcaneus, researchers classified all 71 patients into three types. Three specific types of PCS were noted: type I, angle was less than 30°; type II, angle was from 30° to 60°; and type III, angle was more than 60° (Figures 1 and 2).

### 2.4. Methods for Measuring the PF

**Figure 3:**
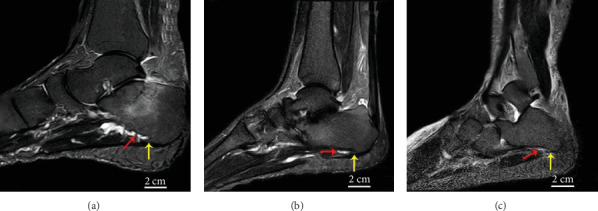
Feet MRI of three patients. The red arrows point to the plantar fasciitis, and the yellow arrows point to the plantar calcaneal spur.

Feet MRI scans of all participants were obtained. Then, researchers used images to judge the MRI grade of each patient (Figure 3, Table 1). Observation was carried out simultaneously by two medical practitioners working for more than 10 years; if there was any difference in the results, the third medical practitioner was responsible for the judgment.

The researchers assessed the grade of PF according to the ratio between the edema width and the total plantar fascia width on selected sagittal imagines. (Grade normal: the ratio was less than a quarter. Grade mild: the ratio was between a quarter and a half. Grade moderate: the ratio was between a half and three quarters. Grade severe: the ratio was more than three quarters).

### 2.5. Methods for Getting Visual Analog Scale (VAS) Scores

All patients completed the visual analog scale test to record their preoperative pain. At 12 months of follow-up, researchers had patients finish the VAS test to record the pain of patients (Table 2).

### 2.6. Methods for Getting American Orthopedic Foot and Ankle Society (AOFAS) Scores

The researchers used a 1994 edition of the American Orthopedic Foot and Ankle Society (AOFAS) scoring system to rate the patients. The higher number refers to a better foot function. All patients got AOFAS ankle-hindfoot scores to evaluate the foot function before removing the PCS. At 12 months of follow-up, they did this test to assess the foot function index again (Table 2).

### 2.7. Methods for Surgical Removal of Calcaneus Spurs

Firstly, we used nonsteroidal anti-inflammatory drugs (paracetamol, qid, 150 mg/d) to patients. Secondly, patients were not only asked to adjust the heel pad to reduce pressure but also asked to do stretching exercises daily. Finally, surgeons would operate on patients who did not respond to medical treatment after three months and had a VAS score of over 5.

**Figure 4:**
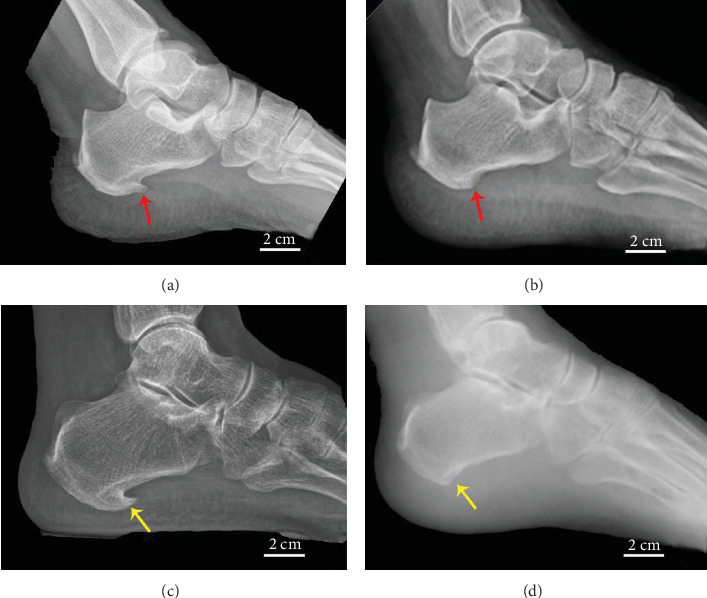
Before the removal of PCS in patient no. 1 (a) and after the removal of PCS in patient no. 1 (b); before the removal of PCS in patient no. 2 (c) and after the removal of PCS in patient no. 2 (d). The PCS of patient no. 1 (red arrow) and the PCS of patient no. 2 (yellow arrow).

The operation used a deep fascial approach, performing endoscopically. The patient was placed in the supine position with a cushion placed under the ankle to facilitate external rotation of the ankle. Routine intraspinal anesthesia was used. After successful anesthesia, a routine sterilized towel was used, a blood-expelling band was used, and a tourniquet was pressed 0.03 MPa. A 3-5 cm transverse incision was made on the lateral side of the lower margin of the calcaneus. The skin and subcutaneous tissue were cut layer by layer to reach the PCS. Release the adhesive soft tissue and remove the increased bone from the base of the calcaneus with a small burr (Figure 4). After releasing the tourniquet and carefully stopping the bleeding with the electric knife, touch again to check for any residual PCS. If there were no residual bone spurs, the subcutaneous tissue and skin were sutured layer by layer after rinsing; once the bandaging was completed, lift the patients' limb to reduce swelling.

### 2.8. Methods for Patients' Foot Function Recovery

One to 3 days after the operation, the ankle and toe were given functional exercise, supplemented by physical therapy such as ice compress. The affected limb was supported to walk with a load gradually for two weeks after the operation. The patient was trained with a completely normal load gradually for 3 to 4 weeks.

### 2.9. Statistical Methods

All measurements were expressed as mean and standard deviation (SD). Categorical variables were described by number and percentages. The homogeneity of variance was performed by using the Shapiro-Wilk test. One-way ANOVA was used to compare 3 types of PCS considering a *P* value < 0.05 as statistically significant. Fisher's exact test was used to compare the MRI grade differences from preoperative to 12 months postoperative. IBM SPSS Statistics, version 17.0 (Chicago, IL), was used for all statistical analyses.

## 3. Results

### 3.1. VAS and AOFAS Scores

According to Table 2, type II (29, 40.8%) had the highest incidence in Chinese population, followed with type I (24, 33.8%). Obviously, type III (18, 25.4%) had the lowest proportion in Chinese citizens.

Preoperative VAS scores showed that type II (7.72 ± 1.10) was significantly higher than type I (6.08 ± 0.93) and type III (5.444 ± 1.0416) (*P* < 0.001). After treatment, postoperative VAS scores of all three types decreased significantly than that of the preoperative group (*P* < 0.001).

At 12 months postoperatively, firstly, Table 2 shows significant improvements of VAS scores in all three types (*P* < 0.001). Type I had changed from 6.08 ± 0.93 preoperatively to 1.08 ± 0.83 at 12 months postoperatively (*P* < 0.001). Type III had changed from 5.35 ± 1.00 preoperatively to 1.29 ± 0.84 at 12 months postoperatively (*P* < 0.001). Type II had changed from 7.72 ± 1.10 preoperatively to 4.17 ± 1.51 at 12 months postoperatively (*P* < 0.001), which was higher than the other two types (*P* < 0.001). Secondly, AOFAS scores of all three types were significantly improved. The improvements of type I and type III were similar (57.54 ± 4.10 to 84.33 ± 3.09, 56.28 ± 3.43 to 84.00 ± 3.05), but the improvement in type II was smaller at 12 months of follow-up (53.10 ± 4.28 to 78.24 ± 8.57).

### 3.2. The MRI Results

The MRI grade of type I and type III showed statistically significant improvement at 12 months postoperatively (*P* < 0.001), which was not found in type II. Patients' MRI results of the type I PCS group showed that 11 (15.5%) of the 24 feet had no PF, 10 (14.1%) feet were in grade “mild,” and 3 (4.2%) feet were in grade “moderate.” The type II PCS group showed that 2 (2.8%) of the 29 feet had no PF, 7 (9.9%) feet were in grade “mild,” 13 (18.3%) feet were in grade “moderate,” and 7 (9.9%) feet were in grade “severe.” The type III PCS group showed that 12 (16.9%) of the 18 feet had no PF, 4 (5.6%) feet were in grade “mild,” and 2 (2.8%) feet were in grade “moderate”.

### 3.3. Additional Results

Researchers had previously classified PCS into three types based on their length but had not found any statistical difference with VAS scores or MRI grade.

## 4. Discussion

The mechanism of PHP is still unclear. PHP was caused by varieties of physiological factors [7–9]; the most common pathological factor in the population was PF [10, 11]. Research studies on the PF and treatments of PF had a long tradition [2, 13, 15, 30]. Histological studies had found that PF had a fascia fragmentation and degeneration [9], which may be caused by PCS at the plantar bottom [19, 20]. This research had classified Chinese PCS into three types to find the relationship between PCS and PF in Chinese population.

In this study, results showed that type II was the most common in Chinese population, followed type I and type III. All patients with PHP had showed significant improvements after surgical removal of PCS. According to Table 2, the mean VAS scores of three types decreased significantly which demonstrated that the clinical outcomes of patients were great (Table 2). Meanwhile, the mean preoperative and postoperative VAS scores of type II were the highest. This phenomenon may be caused by PCS which stretched forward from the point of plantar fascia insertion to within the plantar fascia [31]. The angle between the PCS and the calcaneus of type I was smaller than that of type II, indicating PCS was superior to the plantar fascia insertion. At the same time, the angle of type III was bigger than that of type II, indicating PCS was inferior to the plantar fascia insertion. More severe PF and more severe pain may be due to the contact of PCS with plantar fascia.

At 12 months of follow-up, all three types had better outcomes after PCS removal. Patients showed significant improvement in both pain and foot function which was demonstrated in this study. Nevertheless, in the type II follow-up, patients' pain index and their foot function did not improve as much as the other two, which suggested that clinicians need take more medical treatment to improve the prognosis when treating type II patients.

All patients with PHP showed a significant improvement in their clinical outcomes after surgical operation, which means removal of PCS was a useful way to reduce the patients' pain in a short time. However, MRI results of type II and VAS scores showed that there were signs of recurrence of PF at 12 months postoperatively (Tables 1 and 2). For type I and type III, the prognosis of surgical treatment was excellent and there were no signs of recurrence of PF at 12 months postoperatively (Tables 1 and 2). So patients with type II PCS need follow-up medical treatment after surgical treatment to improve their prognosis.

Although the prognosis of type I and type III after surgery was similar, the morphology of type III was more specific. The special shape of type III was more likely to damage the plantar soft tissue and plantar fascia. The tip of the PCS pointed in the direction of the plantar was a potential threat to the plantar soft tissue and plantar fascia. Type III patients need more concern according to this situation.

## 5. Conclusion

By comparison and analysis, researchers found that patients with PCS and PHP were suitable for surgical treatment, which would reduce their pain immediately. Patients with PCS who showed no pain did not require operation. For patients with type II PCS, doctors should give them appropriate medical treatment after removal of PCS, which would significantly improve patients' prognosis and reduce their cost of secondary care.

In this research, researchers had proved that type II was more likely to cause severe PF. They also found that the severity of PF and pain were independent of the length of PCS.

## Figures and Tables

**Table 1:** Comparison of the PF MRI grade of preoperative and 12 months postoperative in different types of PCS ($\bar{x}\pm s$).

	MRI grade	Preoperative (*n*)	12 months postoperative (*n*)
Type I	Normal	0	11 (15.5%)^∗^
	Mild	10 (14.1%)	10 (14.1%)^∗^
	Moderate	10 (14.1%)	3 (4.2%)^∗^
	Severe	4 (5.6%)	0^∗^
Type II	Normal	0	2 (2.8%)
	Mild	4 (5.6%)	7 (9.9%)
	Moderate	12 (16.9%)	13 (18.3%)
	Severe	13 (18.3%)	7 (9.9%)
Type III	Normal	0	12 (16.9%)^∗^
	Mild	9 (12.7%)	4 (5.6%)^∗^
	Moderate	8 (11.3%)	2 (2.8%)^∗^
	Severe	1 (1.4%)	0^∗^

^∗^ *P* < 0.05 vs. preoperative. ^∗^Fisher's exact test.

**Table 2:** Comparison of the VAS and AOFAS scores of preoperative and 12 months postoperative in different types of PCS ($\bar{x}\pm s$).

		VAS scores		AOFAS score	
	No.	Preoperative	12 months postoperative	Preoperative	12 months postoperative
Type I	24 (33.8%)	6.08 ± 0.93b	1.08 ± 0.83ab	57.54 ± 4.10	84.33 ± 3.09ab
Type II	29 (40.8%)	7.72 ± 1.10	4.17 ± 1.51a	53.10 ± 4.28	78.24 ± 8.57a
Type III	18 (25.4%)	5.35 ± 1.00b	1.29 ± 0.85ab	56.28 ± 3.43	84.00 ± 3.05ab

^a^ *P* < 0.05 vs. preoperative. ^b^*P* < 0.05 vs. type II.

## Data Availability

Data are available from Lei Zhang (email: zhanglei870722@126.com) for researchers who meet the criteria for access to confidential data.

## Additional Points

*Limitation*. There may be errors in the measurement of the angle and length during X-ray imaging. This study explored the differences between different types of PCS, without exploring the differences between the sexes. Surgical treatment was not included in the comparison of asymptomatic patients.
